# Citational politics in and through animal geographies: interrogating onto-epistemological diversity

**DOI:** 10.1080/14702541.2024.2374375

**Published:** 2024-07-11

**Authors:** Kathryn, Krithika, Rosemary

**Affiliations:** a Independent Scholar; b Institute of Geography, University of Edinburgh, Edinburgh, UK; c Department of Geography, Simon Fraser University, Burnaby, Canada

**Keywords:** Citation politics, equity, diversity and inclusion, onto-epistemological trends, animal geographies, anthropocentrism

## Abstract

This paper contributes to geographic literature on the effects of inequity in citational practice and politics, focusing in particular on onto-epistemological diversity (or lack thereof) in animal geographies’ citational structures. Through a bibliometric analysis of journal articles in Anglophone animal geographies (as a subdiscipline of human geography), we examine the intersections between citational trends, the contours of knowledge in the field and everyday academic lives. Our goal in this paper is to highlight some of the ways in which citational inequities are fueled. Specifically, our analysis shows that within Anglophone animal geographies, citational esteem can accrue through institutional networks and shared onto-epistemologies, which often go along with ethical and political orientations that refrain from explicitly contesting the status-quo of anthropocentrism. We ground our analysis with a reflective discussion of everyday academic practice to understand the multi-scalar dynamics and implications of citational politics and prompt heightened reflexivity among geographers concerning how animal and other geographies are constructed and reproduced – and how these reproductions can be contested.

## Introduction

This paper has its roots in conversations among the three of us on what equity, diversity, and inclusion (EDI) might mean in our own geographical scholarship and practice. While EDI issues have not been the focus of our research, we have each engaged with them in our everyday academic practice, including the question of citational politics. Our interest in citational politics comes both from personal experiences of citation and from a broader concern about what we identified as potentially troubling trends in the citational structures of our home field of Anglophone animal geographies, a subdisciplinary field of human geography. We set out researching, thinking, and writing for this paper because we wanted to know *whose* work is being cited, why, with what personal and political stakes, what this says about the field, and what this means for crafting inclusive and diverse scholarship.

The paper joins conversations about issues of exclusion in the university – from cultures of whiteness in institutions and curricula to gender and racial bias in teaching evaluations and citation. Over the past forty years or so, EDI initiatives and principles have proliferated in universities around the world, in response to much longer-standing demands from decolonial, anti-racist and feminist movements, among others, by groups of people who have been systemically discriminated against and excluded from university spaces. Although there is no one approach to EDI, for us, the core of EDI within the academy is a recognition of (1) the value of a diversity of experiences, identities and knowledges for universities and scholarly inquiry, and (2) how racism, patriarchy, colonialism, ableism and other systemic oppressions, including anthropocentrism, shape universities, people’s experience and opportunities in universities, and the contours of knowledge that are produced and legitimated. EDI is – or should be – an active working for diversity and against those systems of oppression and their manifestation in the academy.

At the same time, EDI initiatives have been criticized for their failure to bring about real change (Ahmed, 2012; Henry et al., 2017). Specific to this paper’s inquiry, as individual academics, we continue to encounter varying manifestations of worrying citational politics in our research and teaching in animal geographies. As we will discuss, it was not easy or straightforward to make connections between broader debates on citational inequities and our own academic practice. Spurred by a sense of disappointment that a relatively young field such as animal geographies should replicate the problems and exclusions that have been challenged previously, and intrigued by the difficulties in identifying the links between wider issues and everyday scholarship, we set out to investigate the pervasiveness of exclusionary citational politics.

For this, we turned to Anglophone animal geography to examine publishing trends. We modeled our investigation on parallel analyses of other subdisciplines and themes in geography, including economic geography (Rosenman et al., 2020) and neoliberal natures (Apostolopoulou et al., 2021; Bigger & Dempsey, 2018). We take direction from scholars interrogating taken-for-granted structures of colonialism, whiteness, and patriarchy in the academy (Alderman et al., 2021; Daigle, 2019; Eaves, 2021; Henry et al., 2017; Kobayashi & Peake, 2000; Mahtani, 2004, 2006, 2014; Oswin, 2020; Pulido, 2002; Todd, 2015, 2016), especially those concerned with citation politics. Like this other work on citational politics in various subdisciplines of geography, we use animal geographies to illustrate what are more broadly relevant points about citational structures in human geography at large.

Here, we note that our analytical focus on Anglophone animal geographies might lend itself to the misinterpretation that Anglophone institutions are where animal geographies are being produced. It is important to clarify that this is not the case: language barriers on the part of scholars who may not speak a particular language, as well as distributional access to non-English language journals and publications, may affect in significant ways how non-English publications get included – or more so, not – in Anglophone scholarly debates.

Although there are many different forms of diversity that constitute (or are excluded from) disciplines, in this paper we focus on two forms of citational diversity that were noteworthy findings in our bibliometric analysis. First, we consider the geographic diversity of articles and in relation to citation. While geographic diversity can refer to many aspects of published work, such as field site location, we here specifically address *where* authors are located institutionally. Second, we inquire into the onto-epistemological diversity of articles in relation to citation, by which we mean diversity in ways of knowing and thinking, especially in terms of the published articles’ conceptual frameworks and their political implications.

Inevitably, these lines of investigation led to uncomfortable questions about our own work and positionalities. As university-affiliated scholars – one a woman of color from the Global South and two white women from the Global North – who have been publishing in Anglophone animal geographies for a decade or more (from the Global North), we are fully implicated in our study, as both citers and cited. This sense of discomfort prompted us more to closely examine how broader/structural citational inequities and reform operate at the personal level, at the level of everyday academic practice. Bringing together our own bibliometric study with autoethnographic reflections on our journeys through these matters, this paper attends to the interplay between the personal and political. By teasing out some of the lived realities of subdiscipline-level citational trends, our aim is to illuminate the varied manifestations and multi-scalar implications of citational politics, and to generate momentum for concerted action at multiple scales and spaces.

In what follows, after discussing well-developed debates on citational politics, we report the results of our bibliometric study. The results suggest that citational inequities can manifest in multiple ways. Specifically, our analysis highlights the importance of paying attention to how citational esteem accrues to groupings of scholars who are networked institutionally and share onto-epistemological orientations, thereby shaping the very contours and tenor of what is considered valuable scholarship. Our bibliometric analysis focuses on the ‘top 25’ cited papers in the field of animal geography, something we recognize reproduces citations of certain scholars and thus contributes in part to the very problem we are aiming to address. However, we have chosen to take this approach because, in order to critique the nature of citation in the field, we have to first identify the object of our critique to understand the particulars of who is being cited, where they are, and why. Partially to redress the problem of reproducing existing citational structures, we list these ‘top-cited’ works only in the body of the paper, treating them as a dataset (as opposed to scholarly citations that would be listed in the bibliography). The only exceptions are two articles (Buller, 2015; Sundberg, 2014) whose substantive content we draw upon in our discussions, independently of their presence in our dataset.

Building on the albeit fraught origin and nature of our analysis and findings, we go on to consider the implications of these citational structures – a politicization of the personal and day-to-day practice of citation – foregrounding the difficulties in translating theorization about citational politics into action and, especially, in driving ethically oriented scholarship for animals themselves.

## Diversity and the politics of citation

Citation is not a neutral, self-evident, meritocratic practice; it is a powerful technology for reproducing (or contesting) disciplines, including the scholars and ideas positioned at their fore – what Sara Ahmed (2013) calls ‘citational structures.’ Citational practices shape what and whose scholarship is included (or not) in scholarly conversations, and what and whose scholarship is held in esteem and thereby ‘able to set the terms of the debate’ in a field (Mott & Cockayne, 2017, p. 961). Citation thus shapes the academy itself, both in terms of its membership and the knowledges it produces.

Three interlinked aspects of diversity are particularly relevant to citation practices: socio-demographic, geographic and onto-epistemological. It is challenging if not impossible to land on terms that capture what are complex and overlapping terrains of difference; these three terms are imperfect, but are a necessary means of conducting, interpreting, and considering the implications of an empirical study of citational structures. By socio-demographic diversity, we mean *whose* work is cited: i.e. how axes of demographic/social difference such as gender, race, nationality, and ability are represented in a field. Studies on the socio-demographics of citation consistently demonstrate, for instance, a gendered and racialized bias in citation (Ahmed, 2012; Dion et al., 2018; Henry et al., 2017; Ray, 2018). Our bibliometric analysis does not allow us to observe gendered and racialized patterns in citation without reproducing problematic practices of inferring others’ identities from, say, names or photographs (see Teele & Thelen, 2017).

We are able, though, to track a second, related aspect of diversity: geographic diversity, specifically, *where* the people who are cited are based: i.e. the distribution of scholars’ institutional locations. Socio-demographic diversity and geographic diversity are distinct but could shape each other, for example, along lines of nationality; i.e. authors are more likely to be based in an institution in the same country as their nationality. However, we do not examine the links between the two in our analysis.

Finally, onto-epistemological diversity pertains to *what* work is cited: the character of knowledge that circulates within a field, especially in relation to what is considered legitimate and important; what types of knowledge are overlooked or considered irrelevant, overly particular, illegitimate, or of inferior quality; and the socio-political contexts within which these knowledges are generated. This *what* of the work, as we find, is shaped in large part by the *who* (which scholars are deemed ‘top’) and *where* (which places have a concentration of scholars producing work in English from Anglophone institutions). Our bibliometric analysis thus considers the onto-epistemological orientations of the top-cited work.

These three forms of diversity are entangled and highly contested terrains of difference in citation politics and broader knowledge politics at universities worldwide. Citation politics are part of broader contemporary movements at universities to contest and expand the narrow range of perspectives and limited pool of ‘experts’ in academic knowledge production. From the Rhodes Must Fall protest movement that began in South Africa and spread globally in 2015 (Ahmed, 2020) to Black Lives Matter protests on campuses across the US and around the world (Douglas et al., 2020), students, Black scholars, scholars from the Global South, Indigenous scholars, scholars of color and their allies are leading vibrant movements for decolonization and Indigenization and against racism, especially anti-Black racism, at universities around the world. These movements sit within a centuries-long history of critical scholars criticizing and struggling against the deeply grooved Eurocentrism and whiteness of academic knowledge production (see references cited earlier).

The lack of socio-demographic, geographic and onto-epistemological diversity against which these movements push has powerful implications for academic knowledge production at multiple scales, from individual scholars to global fields of study. In terms of citation, all three forms of diversity shape academic fields’ evolution. Socio-demographically narrow citation patterns limit the range of perspectives and approaches that circulate in a field, thereby shaping research and teaching in restrictive ways and impoverishing what could be a richer discipline. In turn, dominant onto-epistemological trends restrict the space available for fresh and innovative scholarship by those who are not already established as well-cited ‘experts’. The location of ‘expertise’ within disciplines usually reflects and reinforces wider socio-demographic power structures – what is circulated as high-quality knowledge in any field is often produced by a small pool of ‘experts’, often from privileged socio-demographic and institutional backgrounds. Structurally, this situation is reinforced by the nature and power of search engines and their inclusions and omissions based on quantitative tracking, paywalls where institutional journal subscriptions shape the available scholarship, and the education of new scholars in conducting particular kinds of literature reviews.

In a positive feedback loop, citations not only reflect existing power relations but are also themselves power-generating. When we cite something, we give it legitimacy and authority. As Mott and Cockayne (2017, p. 964) put it, ‘through the process of citation, we bring with us those bodies and ideas [and we would add geographies] deemed legitimate and worthy of attention and dialogue – those who we want to remember.’ This is often irrespective of how we use it in our work; Google Scholar and other citation indices only pick up the number of times something is cited, as opposed to what is said about it. This legitimacy is self-perpetuating. The more something is cited, the more it is likely to be cited, until it achieves the status of that thing that cannot *not* be cited, regardless of whether the work is meaningful in the context in which it is being cited. Consequently, citation is what Ahmed (2013, n.p.) describes as a ‘reproductive technology, a way of reproducing the world around certain bodies.’ The question is: what worlds are reproduced and how?

In this paper, we begin to answer this question with our focus trained on animal geographies, a subdiscipline of human geography which comes together as an academic field through the constitutive practices of knowledge-making and – sharing. Citation operates in this disciplinary formation as a key technology of power, reproduction, repetition and resistance (Ahmed, 2013; Mott & Cockayne, 2017). As Ananya Roy (2020, p. 20) writes, ‘[c]itationary practice is a cornerstone of the self-narration of disciplines as well as of academia’s respectability politics. It is a key mechanism through which we discipline ourselves, offer up obedience, and maintain hierarchy.’ This is a core feature of academic training. New geographers are brought up through higher education to appreciate and become fluent in particular kinds of knowledges, learning to cite and assign hierarchized systems of value to certain scholars and works. Examinations in graduate education in many parts of the world are, in fact, precisely organized around this disciplinary technology and gatekeeping – students are expected to know, have read, and engaged with the Big Names in order to achieve PhD candidacy.

While our focus here is on journal publishing, citational disciplining extends beyond publishing and bibliographic creation in written texts. Citational practices also include speaking invitations, references in casual conversations, inclusion in syllabi and other forms of affirmation (Ahmed, 2017, p. 148). This broader realm of citational practices and the particular contexts in which they manifest mutually reinforce one another. Someone who is written about, taught, talked about and invited to give talks ends up being written about, invited to give talks, taught and talked about more, solidifying a ‘star’ status – amplifying the visibility of that individual and their work. This in turn reinforces citational inequities and (in)visibility at the personal level – the less cited you are, the less cited you are likely to remain and *vice versa* – as well as impacting the contours of the field itself, which tends to evolve in the direction of the approaches and ideas contained in the writings of those who are highly cited (Ray, 2018).

Given these stakes, an investigation of how citational politics manifests on the ground, shapes, and is shaped by everyday academic practice becomes crucial. This kind of analysis necessitates heightened reflexivity concerning how, for whom, by whom and to whose benefit academic disciplines are constructed and reproduced. In particular, our paper builds on and reaches beyond the extensive scholarship on citational politics *vis-à-vis* people (e.g. scholars and authors) to examine their ethical and political ramifications for the very subject matter of animal geographies, animals themselves.

## Animal geographies’ citational structures

In her 2016 *Progress* report on animal geographies, Alice Hovorka presents a case for globalizing and decolonising the subdiscipline. She surveys the wide range of geographic locations where animal geographers’ fieldwork is based in order to illustrate the global scope of animal geographies as a *globalizing* discipline. While the survey reports an impressive array of places around the globe that serve as sites for animal geographies, emphasizing too the plurality of multispecies relationships unfolding in different locales (also see Gibbs, 2020), most of the authors mentioned are located in the Global North (primarily in North America and the UK). This means that while knowledge is being produced *about* human-animal relations in a range of different places, knowledge *from* a wider diversity of places is not being well-represented in Anglophone scholarship – suggesting not that this scholarship is not being produced in non-Anglophone locales, but that it is not being accessed and cited in Anglophone scholarly discussions and debates. Despite the global nature of research sites, this collection of scholarship reflects to a certain extent what Hovorka and others have identified as the ‘predominantly white, Anglophone and Western origins and trajectories of animal studies scholarship’ (Hovorka, 2017, p. 2; Sundberg, 2014). In and beyond geography in the past decade, social science scholars have accordingly shone a spotlight on exclusions in the ‘animal turn’, especially critiquing the erasure of Indigenous and Black theory and knowledge from animal studies and a wider universe of posthumanism, including the conflation of race and species in this scholarship (Ahuja, 2009; Belcourt, 2015; Jackson, 2015; Kim, 2018; Sundberg, 2014; Todd, 2015, 2016), as well as the role that gender plays in animal studies publishing (Fraiman, 2012; Probyn-Rapsey et al., 2019).

Furthermore, the location from which scholars conduct the majority of their work (e.g. where their home institution is based) is constitutive of their work, yet often goes unacknowledged. Juanita Sundberg (2014, p. 36) critiques this lack of attention to location. She writes that ‘silence about location is a significant performance that enacts Eurocentric theory as universal, the only body of knowledge that matters.’ Coupled with a parallel silence ‘about Indigenous scholarship … ., these maneuvers perpetuate colonial violence’ (p.36). Consequently, Sundberg highlights the significance of acknowledging *place* in decolonizing posthumanist theorizing, following from the epistemic importance in Indigenous scholarship of place-based specificity and non-universalizing claims. It therefore becomes important to ask not only where are animal geographers writing *about*, but also *from where* are they writing? This *where* can be understood as the geographic place or institutional location about/from which the author is writing, as well as the onto-epistemological roots within which the author’s thinking and writing is situated.

We pick up on Hovorka’s (2017) and Sundberg’s (2014) observations about the Eurocentric and Anglophone trajectories of animal geographies to investigate how citational inequities operate *within* the already limited terrain of Anglophone animal geographies. In other words, we ask whether citational discrimination can take forms *beyond* well-documented socio-demographic axes. Specifically, we ask: what sorts of knowledge are being circulated and reinforced within Anglophone animal geographies? In which geographies, epistemologies and theories are they situated? And what are the ethical and political implications for animals themselves?

### A bibliometric inquiry

To take a step toward addressing these questions, we undertook a bibliometric study of publishing trends in Anglophone animal geographies from 1998 to 2019, followed by an inductive content analysis on the top 25 cited articles. Why 1998 as a starting point? That year, Jody Emel and Jennifer Wolch published *Animal Geographies*, the first edited volume on the at-the-time emerging ‘third wave’ of animal geography as an area of study in human geography. This ‘third wave’ was distinct from long-standing physical geography and cultural ecology studies of animals in its central concern for how spatial forms shape human-animal relations and *vice versa* and, importantly, its treatment of animals as *subjects* of their own lives (Hovorka, 2018; Urbanik, 2013).

In the following two-plus decades, the number of animal geographers and their publications grew quickly, as our bibliometric study shows, and the subdiscipline became formalized in scholarly associations, research clusters, encyclopedia and handbook entries (Lorimer & Srinivasan, 2013; Wilbert, 2009) and *Progress* reports (Buller, 2014, 2015, 2016; Gibbs, 2020, 2021; Hovorka, 2017, 2018, 2019). In recent years, some animal geographers have pushed the field to ‘extend beyond’ animals to the more-than-human more broadly, including ‘nonhuman, lively and inert materials, elements, forces and institutions that shape animals’ lives and human–nonhuman-animal relations’ (Bear, 2021; Gibbs, 2020, p. 774). We proceed with this broader definition of animal geographies or the more-/other-than-human here, in part because of the difficulty drawing a line between animal geographies research and more-than-human research in many cases, as we explain below.

To gather the data, we used Web of Science (WoS). Like all databases, WoS provides only a partial picture of scholarship in the field and has varied limitations. Perhaps most importantly, books are not included – an important omission in a subfield like animal geographies, which is arguably in part founded on two edited books (Wolch and Emel, *Animal Geographies: Place, Politics and Identity in the Nature-Culture Borderlands*, 1998; Philo and Wilbert, *Animal Spaces, Beastly Places: New Geographies of Human-Animal Relations*, 2000). These books’ exclusion from our analysis is not a reflection of their stature in subdiscipline; our paper is not a full review of the field. Instead, we use articles as a window into the operation of citational politics. Neither are all journals included in WoS. Most notably for our purposes *Capitalism Nature Socialism* (*CNS*) and *Environment and Planning E: Nature and Space* (*EPE*) are not in the database, but, because animal geographies work in *CNS* is rare and *EPE* only began publishing in 2018 (and our dataset ends in 2019), we do not think the omission of these journals overly limits our analysis. Two other limitations must be noted: first, the search terms and filters used in WoS also shape the results that are generated, much like with statistics of any kind; for example, the results of our search include self-citations while not capturing incorrect citations (for instance, through misspelling less common surnames); second, WoS indexes primarily Anglophone scholarship and as such precisely reproduces the problems noted by others such as Hovorka (2017) and Sundberg (2014).

However, our analysis focuses on citational inequities *within* Anglophone animal geographies, and *beyond* socio-demographic diversity. On the whole, while databases such as WoS are replete with problems in how they aggregate scholarship, these are databases that are commonly used to find literature, typically through search methods that are not fine-tuned to the technicalities of how the database is constructed. Therefore, analysing publishing trends through them, using search methods that are undeniably imperfect, helps to develop a grounded picture of the issues at stake in everyday academic practice.

In WoS, we searched all Anglophone journals that list geography as a key field (52 journals), which meant we excluded journals like *Animal Studies Journal*, *Humanimalia*, *Politics and Animals* and *Society and Animals*, in which animal geographers do publish. The search of paper titles, abstracts, and keywords was conducted using a suite of search terms: animal*, *more-than-human*, ‘*more than human*,’ *other-than-human*, ‘*other than human*,’ anthropocentri*, *non-human*, ‘*non human*,’ posthuman*, ‘post-human*,’ *multispecies*, speciesis*.^1^ This original search yielded 978 articles. 540 articles were subsequently removed from the dataset based on manual clean-ups (in some cases involving searching full papers) to ensure that each article is broadly related to animal geographies, which we defined as concerning all living beings, including plants, to reflect the broader, more-than-human approach to animal geographies mentioned earlier. This does mean that our dataset includes subfields like plant geographies and hybrid geographies, which have somewhat different intellectual lineages than animal geographies, but there is fruitful crossover and conversation between these domains that we wanted to capture in our dataset. Indeed, the crossover is at times so strong that it would have been difficult to determine whether some papers in our dataset belonged in the stricter animal geographies pile or the broader more-than-human pile. We did, however, remove papers from the dataset if they entailed physical geography addressing living things or if they did not centrally engage nonhuman life in the analysis. The final dataset consists of 437 articles.

**Figure 1.**
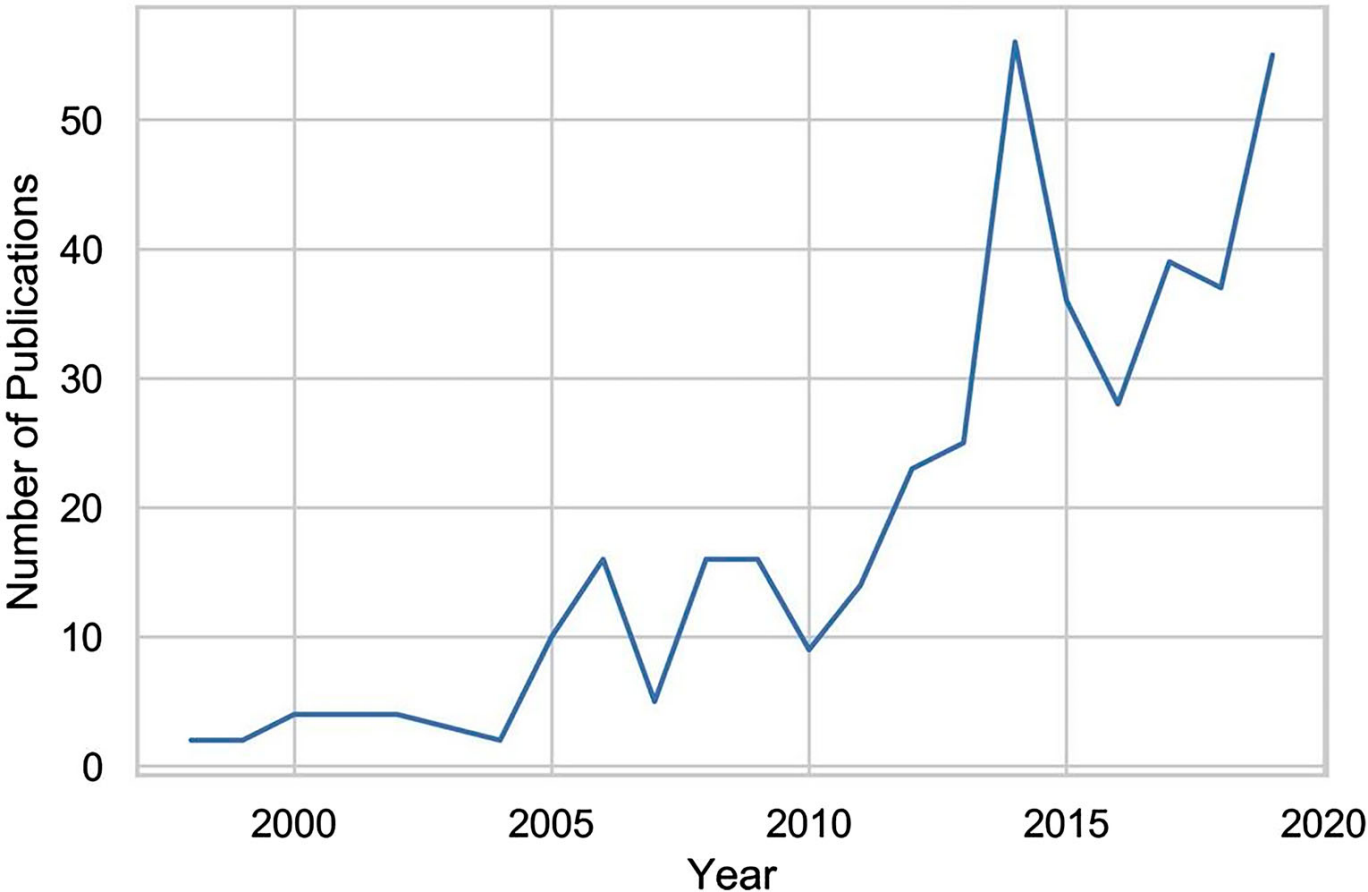
Number of animal geography publications, 1998–2019. (Source: Authors).

The bibliometric analysis shows speedy growth in animal geography publications (see Figure 1). From the late-1990s until 2005, fewer than 10 animal geography articles per year were published in the geography journals included in our analysis. After 2005, despite some variability from year to year, an upwards trend has meant that over the past 10 years an average of 32 animal geography articles per year have been published.

The majority of this growth has come out of the UK. An analysis of the location of the first author shows that papers authored by UK-based scholars comprise 41 percent of the total dataset. USA-based authors make up 19 percent, authors in Australia 14 percent, and authors in Canada 10 percent. The remaining 16 percent of the total dataset is comprised of 26 other countries, including, in order of prominence: Finland, New Zealand, Norway, Sweden, Singapore, Germany, Belgium, Austria, Denmark, the Netherlands, Spain, Chile, Estonia, France, Hong Kong, India, Ireland, Italy, Japan, Poland, Portugal, Romania, Slovenia, South Africa, South Korea, and Switzerland.

The data also show that the top-cited authors are based in the UK, Australia and Canada (see Table 1), all English-speaking countries where the privileges of publishing in English serve scholars well. Authors’ individual citation counts are determined by average citations per year, from the year of each author’s first publication. To calculate the average, we added each author’s total citations per paper and divided the total by the number of years since the author’s first publication. For example, if an author published their first paper in 2015 and another paper in 2017, and the first paper was cited 100 times and the second 20 times, the author’s average citations per year up to 2020 become (100 + 20) / 5 = 24. Of the top 25 most-cited authors, 17 are based in the UK (68 percent), five in Australia (20 percent) and three in Canada (12 percent).

**Table 1.** Top 25 most cited authors (by average citation per year from date of first publication).

Author	Location
Helen Wilson	UK
Juanita Sundberg	CAN
Maan Barua	UK
Steven Hinchliffe	UK
Sarah Whatmore	UK
Rosemary Collard	CAN
Chris Bear	UK
Jamie Lorimer	UK
Lewis Holloway	UK
Emma Power	AUS
Matthew Kearnes	AUS
Monica Degen	UK
Jessica Dempsey	CAN
Jennifer Atchison	AUS
Gareth Enticott	UK
Henry Buller	UK
Carol Morris	UK
Hayden Lorimer	UK
Krithika Srinivasan	UK
Timothy Hodgetts	UK
Lesley Head	AUS
Sally Eden	UK
Mara Miele	UK
Franklin Ginn	UK
Bawaka Collective	AUS

Souce: Authors.

If we zoom in on the top 25 most-cited animal geography articles in our dataset, by total citation, again all articles are authored by scholars in the same locations as above, with the addition of USA representation, which now accounts for four of the top 25 (see Table 2). We elect to present the top-cited articles by total citation as opposed to average in order to convey which articles have achieved the most circulation over time – a proxy for their overall influence in the field over time. The pitfall of this approach is of course that older articles have higher citation counts by virtue of having been in circulation for longer, although, as Table 2 shows, there are also some more recent (e.g. 2017) articles high in the list.

**Table 2.** Top 25 cited articles by total citations.

Author-date	1st author location	Article title	Journal	Citations
Hinchliffe et al. (2005)	UK	Urban wild things: a cosmopolitical experiment	*EPD: Society and Space*	271
Lorimer H (2006)	UK	Herding memories of humans and animals	*EPD: Society and Space*	184
Sundberg (2014)	CAN	Decolonizing posthumanist geographies	*cultural geographies*	142
Wolch (2002)	USA	Anima Urbis	*Progress in Human Geography*	124
Whatmore and Thorne (1998)	UK	Wild(er)ness: reconfiguring the geographies of wildlife	*Transactions of the Institute of British Geographers*	122
Hinchliffe (2001)	UK	Indeterminacy in-decisions: science, policy and politics in the BSE (Bovine Spongiform Encephalopathy) crisis	*Transactions of the Institute of British Geographers*	121
Lorimer J (2010)	UK	Moving image methodologies for more-than-human geographies	*cultural geographies*	120
Fox (2006)	UK	Animal behaviors, post-human lives: everyday negotiations of the animal-human divide in pet-keeping	*Social and Cultural Geography*	117
Robbins (2006)	USA	The politics of barstool biology: Environmental knowledge and power in greater Northern Yellowstone	*Geoforum*	113
Bingham (2006)	UK	Bees, butterflies, and bacteria: Biotechnology and the politics of nonhuman friendship	*EPA: Economy and Space*	110
Wilson (2017)	UK	On geography and encounter: bodies, borders, and difference	*Progress in Human Geography*	106
Ogra (2008)	USA	Human-wildlife conflict and gender in protected area borderlands: a case study of costs, perceptions, and vulnerabilities from Uttarakhand (Uttaranchal), India	*Geoforum*	96
Hobson (2007)	AUS	Political animals? on animals as subjects in an enlarged political geography	*Political Geography*	95
Power (2008)	AUS	Furry families: making a human-dog family through home	*Social and Cultural Geography*	94
Convery et al. (2005)	UK	Death in the wrong place? emotional geographies of the UK 2001 foot and mouth disease epidemic	*Journal of Rural Studies*	89
Nally (2011)	UK	The biopolitics of food provisioning	*Transactions of the Institute of British Geographers*	85
Panelli (2010)	UK	More-than-human social geographies: posthuman and other possibilities	*Progress in Human Geography*	85
Johnson (2008)	UK	Beyond the clearing: towards a dwelt animal geography	*Progress in Human Geography*	85
Holloway (2007)	UK	Subjecting cows to robots: farming technologies and the making of animal subjects	*EPD: Society and Space*	83
Collard et al. (2015)	CAN	A manifesto for abundant futures	*Annals of the Association of American Geographers*	82
Power (2005)	AUS	Human-nature relations in suburban gardens	*Australian Geographer*	82
Sundberg (2014)	CAN	Diabolic caminos in the desert and cat fights on the Rio: a posthumanist political ecology of boundary enforcement in the US-Mexico borderlands	*Annals of the Association of American Geographers*	79
Cloke and Perkins (2005)	UK	Cetacean performance and tourism in Kaikoura, New Zealand	*EPD: Society and Space*	78
Watson and Huntington (2008)	USA	They’re here-I can feel them: the epistemic spaces of Indigenous and Western Knowledges	*Social and Cultural Geography*	77
Buller (2015)	UK	Animal geographies II: methods	*Progress in Human Geography*	76

Source: Authors.

Of the top 25 articles by total citation, 15 are first-authored by UK-based scholars (60 percent), four by USA-based first authors (16 percent), three by Australia-based first authors (12 percent), and three by Canada-based first authors (12 percent). Compared to the total dataset, the UK is over-represented in the top 25 articles (60 percent of the top 25 versus 41 percent of the total). In general, in the dataset, locational diversity of authorship decreases with total citation. With the exception of one paper published out of New Zealand (Haggerty and Travis, including a USA-based author), none of the other 26 countries noted earlier (comprising 16 percent of total articles) are represented in the top 50 most-cited articles. As discussed before, this dataset excludes books and book chapters; however, prominent books in the field like Wolch and Emel’s *Animal Geographies*, Philo and Wilbert’s *Animal Spaces, Beastly Places*, and Urbanik’s, *Placing Animals: An Introduction to the Geography of Human-Animal Relations* – cited over 800, 1000 and 300 times respectively, according to another imperfect database, Google Scholar – feature almost exclusively USA – and UK-based scholars, so it is unlikely that the inclusion of these books would significantly alter the geographic distribution.

The most common author-provided keywords for these top 25 articles are *animal geography/ies* (five mentions), *posthumanism* (five mentions), *political ecology* (three mentions), *actor-networks/actor-network theory* (three mentions), more-than-human (geographies) (three mentions), and *animals*, *relational ontologies*, *conservation*, *decolonising/decolonisation* and *anthropomorphism* (each mentioned twice). An additional 76 keywords appear once, suggesting a significant variety of topics and themes in the most-cited animal geographies literature. This confirms animal geographers’ past observations. Ten years ago, Henry Buller (2014, p. 310) described animal geographies as a ‘porous, shifting and eclectic heterogeneity of ideas, practices, methodologies and associations within a more-than-human life/world.’ For Leah Gibbs (2020), this diversity has only expanded in recent years, although she notes an important empirical bias towards terrestrial mammals. This topical diversity nonetheless somewhat obscures prominent patterns regarding themes and theoretical approaches in the top-cited literature.

### Interpreting the bibliometrics

A close reading of the articles in Table 2 yields a finer-grained sense of these themes. The most prominent conceptual theme across the articles is broadly human-nonhuman relationality. Under this theme, several articles focus on unsettling the human-animal divide and developing an understanding of relational agency – i.e. the capacity of nonhumans to shape (and be shaped by) everything from technologies and commodification to geopolitics and gardens, if always in concert with other beings. Given the prominence of the theme of relationality, it is perhaps unsurprising that Actor-Network Theory (ANT) is the most widely used theoretical approach across these articles, found especially in the older articles in the top 25 (e.g. Cloke & Perkins, 2005; Hinchliffe, 2001; Hinchliffe et al., 2005; Hobson, 2007; Johnston, 2008; Panelli, 2010; Power, 2005; Sundberg, 2014; Watson & Huntington, 2008 – with Buller, 2015) being the exception to the older designation, although his *Progress in Human Geography* report on animal geographies is of course largely focused on reviewing past work in the field). Continental philosophers, especially Deleuze and Foucault, also drive several of these earlier top-cited articles (e.g. Panelli, 2010; Johnston, 2008; Bingham, 2006; Hinchliffe et al., 2005; Lorimer, 2010; Watson and Huntington, 2008; Nally, 2011; Holloway, 2007).

Interestingly, several long-standing literatures and theoretical approaches to human-animal relations do not feature in any significant way in the top-cited articles. The most prominent absences that we have noted are, one, feminist animal studies scholarship, and, two, analytical animal ethics and philosophy. These literatures have made substantial theoretical and empirical contributions to the ‘animal question’ from the 1970s onwards, yet are marginal in the top-cited animal geographies work. The omission of feminist animal studies scholarship (e.g. the work of Carol Adams, Maneesha Deckha, Josephine Donovan, Greta Gaard, Lori Gruen, pattrice jones, Marti Kheel, Val Plumwood, and Richard Twine; exceptions in our dataset are Ogra and Sundberg) and its persistent marginalization in animal geographies is consistent with the enduring erasure of the feminist and liberationist roots of animal studies more broadly (Fraiman, 2012). Concerns for and about animals have historically been feminized, and even within the discipline of animal studies where animals *are* taken seriously, work rooted in emotional connection, intimacy, and intersectional approaches to injustice are often sidelined by more ‘rationalist’ theoretical engagement with the topic.

The second absence we see in the top-cited articles is scholarship from analytical animal ethics/philosophy (e.g. the work of Paola Cavalieri, Sue Donaldson, Lori Gruen, Dale Jamieson, Will Kymlicka, Mary Midgley, Martha Nussbaum, Clare Palmer, Tom Regan, Peter Singer, and Gary Steiner; an exception in our dataset is Hobson). This literature and its situation in philosophy is often cast, in our experience, as too ‘animal rights-y’ and hence outside of the purview of geographic engagement. Scholars in these traditions have taken decidedly political, ethically motivated approaches to uncovering *and undoing* the violence that so often characterizes human-animal relations, and so our analysis leads us to believe that these onto-epistemological orientations affect their uptake in animal geographies (more on this later).

In the broader field of animal studies, the last decades have seen the proliferation of journals dedicated specifically to animal studies scholarship – *Hypatia, Politics & Animals, Animal Studies Journal, Humanimalia,* and *Society & Animals,* to name a few. Animal studies scholars, including geographers, publish in these venues, either because they are where animal-related debates are unfolding or because geography journals might be hostile to – or merely deem irrelevant – scholarship on other species. As we reviewed the geography journals where animal geographers have published, we noted the curious absence of a distinctly *animal geographies* journal to serve the discipline, whereas other subdisciplines like *children’s geographies* have developed their own journal. Perhaps it is the absence of a dedicated animal geographies journal that, in part, accounts for the constraints on what kind of scholarship is prized (and possible) within the broader field of geography.

As animal studies scholars, we are left with a choice: publish in animal studies journals where we can engage with other scholars and debates most relevant to our work but which often do not ‘count’ for our careers in the same way, or seek to publish in geography journals where our papers are often outliers. To publish in the latter, animal geographers, ourselves included at every stage of our careers, have to perform certain humanist intentions and contributions in order to be included in the leading geography journals. Junior scholars, in particular, have to conform to geography’s disciplinary norms in order to build their CVs, win jobs and receive the accolades necessary to advance their careers in neoliberal institutions. We have observed that it is often junior scholars (graduate students and recent PhDs) who are doing the most provocative and boundary-pushing work in animal geographies, and therefore, without the venues for publishing, the nature of the field itself remains limited to a certain type of inquiry, even as there is evidence of animal geographies’ objective growth.

Looking at the top 25 articles, a consistent if limited theme is the politics of knowledge, with several authors arguing especially for non-Western ways of knowledge-making in human-animal relations (the papers by Lorimer, Sundberg, Watson, and Huntington). Somewhat surprisingly, few of these top 25 articles are directly focused on making any strong critical or political arguments about existing human-animal relations, especially with respect to advancing an explicitly anti-anthropocentric perspective (exceptions in our database are Collard et al., Power [two papers], Wolch, and Whatmore and Thorne). This paucity is despite what Gibbs (2020, p. 775) identifies as an enduring, important call in animal geographies scholarship for ‘politically-engaged work,’ particularly that which considers animals as ‘vulnerable beings whose vulnerability is often tied to their place(s) in human society’ (Srinivasan, 2016, p. 76; quoted in Gibbs, 2020, p. 775). Our thematic review hence prompts questions around the predominance of certain theorists and conceptual frameworks, as well as their ethical and political orientations toward animals – or lack thereof. More specifically, do the onto-epistemological approaches that are favored in highly cited animal geographical literatures produce analyses that are less likely to be overtly anti-anthropocentric, and thus less attentive to the violent effects of humans’ interactions with animals themselves?

Overall, the bibliometric analysis that we performed in large part confirms what Sundberg and Hovorka identify as an overwhelming Eurocentric dominance in the discipline – at least in the sense of who are most cited as ‘top animal geographers’ and where they are located. The findings of the bibliometric analysis with respect to the location of top-cited animal geographies are to be expected, given that this is a corpus of Anglophone scholarship. What is noteworthy, though, is the predominance of the UK in the top-cited articles, as well as top-cited authors, over other Anglophone countries with robust animal geographical scholarship, such as the USA, Australia and Canada. Furthermore, the imperial and colonial practices of Britain and the USA mean that there are numerous countries where academic scholarship, including animal geographies, is conducted in English (our dataset alone contains 30 countries). So what explains the disproportionate citation of UK-based authors over scholars in other countries where English is widely spoken and used in the academy?

We suggest that this is the outcome of the tendency for scholarship to coalesce in certain geographies where conversations unfold in localized academic contexts, and so we see a cluster of conversations around animal geographies unfolding in the UK where scholars get to know each other, are in the same conversations, share onto-epistemological orientations, and thus cite each other more, driving up citation counts and academic visibility (Janssen et al., 2006; Milard, 2014; Teodorescu & Andrei, 2014). For instance, half of the top 10 most-cited articles are authored by four scholars who have collaborated with each other in some fashion or have institutional links with each other. In the top 10 most-cited authors, four have a history of collaboration and/or institutional links. The same question can be asked not just of the UK, but of the location of top scholars more generally (across the UK, Australia, Canada, and the USA). When collaboration and in-network citation generates itself in this way, it naturally draws attention to those scholars when their citation counts are driven up in-network. Merit does have an important role to play in citation, uptake and visibility. However, what we wish to emphasize is that cross-citations within academic networks can create a cycle where it is necessary and normalized to cite particular scholars, thus expanding their prominence and reach. Equally, the quality and merit of a piece of work alone does not guarantee citation. This is especially the case in the contemporary publishing context of burgeoning volumes of publications which can easily submerge those (authors and works) who don’t have additional pathways (such as institutional esteem and networks) of gaining visibility.

These questions about who is cited, where, and why are sometimes read as innocent, practical questions about merit, publishing trends and citation count, but it is precisely their seeming innocuity that conceals the very political nature of their content. Citation, as a public way of attending to scholars’ epistemological, ontological, and methodological orientations, can be a site of either reaffirming or resisting dominant onto-epistemological and ethical and political orientations. There is no neutral or apolitical bibliographic ground.

Informed by the bibliometric data, we argue for the need to expand understandings of how citational inequities are fueled. Gender, ethnicity, ability, and sexual orientation remain crucial axes along which citational discrimination can manifest. At the same time, our analysis shows that citational inequities can also be driven by academic and/or locational networks coupled with shared onto-epistemologies. These networks can produce a dynamic wherein association with particular scholars or onto-epistemologies can enhance citational esteem, initially through in-group citations and then through wider citation as esteem gathers to groupings of scholars, their onto-epistemologies and their associated ethical and political orientations and commitments – in this case, towards animals. These inequities may not always operate along other axes of socio-demographic discrimination such as gender or ethnicity and are therefore more likely to remain unnoticed or interpreted as the outcome of ‘quality’ (more on this shortly). This state of affairs, in turn, quietly shapes and limits the overall contours of knowledge in the (sub)discipline.

## Citational politics on the ground

In the previous section, we outlined broad trends in animal geography’s citational structures and discussed their implications for the contours of the field – and consequently, for animals themselves. Citations, however, are not only political in how they shape disciplines, fields and wider ethical and political structures, but also in how they affect individual people and how individuals adhere to, reinforce, or resist citational trends and pressures. Through our analysis, academic networks and onto-epistemological and ethical and – political proclivities have emerged as areas that need more scrutiny – and that potentially have individual-level impacts on who and what we cite, and the conceptual and political content of our work. Our own experiences in navigating citational politics illustrate how wider bibliometric patterns and citational structures intersect with individual, personal academic lives.

The three of us are each positioned differently within the academy. Although we are all ‘animal geographers’, we do not share the same institutional positions or subject positions – all of which have bearing on citation. This means that, while we have all navigated the gendered nature of citation, we have unevenly experienced the benefits and/or barriers involved in the academic hierarchy, steady employment, the onto-epistemological frameworks we utilize in our work, and a broader culture of whiteness. We do not, then, speak from a unified subject position, or a comfortable one in relation to citational politics. But our varied experiences with citation in animal geography – which range from observing plagiarism of our work to having to cite certain works because of their citational esteem even if they are not the most relevant to the point we wish to make – have made us collectively curious about the personal and political nature of citational structures.

In what follows, we first discuss some of the personal (at the level of individual scholars) impacts that citational politics can have, exploring the complex links between citational esteem on the one hand, and academic merit, career trajectories and mental health on the other. We then examine the challenges of trying to counter existing citational structures, reflecting on how academic gatekeeping and the volume of publishing can exhaust and overwhelm even the best intentions of individual scholars. Building on this, we make a case for more attention to how peer review processes and (re)training within universities, coupled with a sustained push towards slow scholarship, are crucial for reconfiguring the wider academic structures that are barriers to individual efforts to address citational inequities.

### Not being cited

The three of us set out to research and write this paper with some apprehension. For better or worse, academic citation is personal: who we cite, whether or not we are cited, these are not only reflections of the usefulness of ideas, or even of politics, but also speak to our personal sense of what and who matters, including whether our own scholarship is valued. Citational politics are hard to discuss and write about because we are conscious of their direct implications for our careers and, despite continuous efforts to keep this at bay, our sense of self-worth. It is hard to match discipline-level citational inequities with everyday experiences – to name an incident of ‘not being cited’ or being asked to ‘cite a certain scholar’ as instances of exclusionary citational practice. Indeed, while discussing this paper with a senior scholar-friend, one of us was told that it was only to be expected that certain scholars accrued citational esteem and became academic gatekeeper names, while others remained invisible – that it was part of the academic ‘game’.

This comment shows how difficult it is to disentangle academic quality from citation. Citation is a strange politics: it is full of well-known biases, but the narrative that it is a meritocracy, where the best work is cited the most, and the most-cited work is the best, still prevails and is internalized. Speaking for ourselves, even though we are aware of citational politics, if we are not cited, we typically think it is because we are not good enough. Equally, when we are directed to cite certain scholars, the taken-for-granted message is that it is because their work is of particular significance, while the others that we have already cited are somehow inadequate. Not everyone will share these reactions, of course, although our experience with student supervision suggests that early career scholars, at least, commonly assume that citation is a recognition of scholarly quality.

Citational inequities have implications for individual careers. Citation forms a significant part of universities’ neoliberal metrics of ‘impact’ assessment, which is tied to salary, promotion and academic opportunities at the individual level. We appreciate the irony that even as this article is meant in part to push back against this frenzy of uneven metrics-based valuation, it may in fact be used as documentation of the publishing success of the top scholars in the field for promotional and career-advancing purposes. Citational inequities adversely affect the visibility and uptake of work by individual scholars who do not already enjoy citational esteem, with consequences for their professional trajectories. How often we are cited matters when it comes to promotion, invitations to give talks or be a part of a research team, inclusion in reading lists and teaching syllabi, and, as academics, we have at various points in our careers, been acutely conscious of the professional implications of being cited – or not.

Inequitable citation patterns have other personal impacts. Pressures to publish, to be cited and celebrated, to obtain research funding, and other demands related to an academic career path, drastically impact the mental health of academics. It is estimated that 43% of academics experience mild to severe mental health issues (almost twice the number as in the general population), most common among them anxiety and depression (Doyle & Hind, 1998; Gorczynski, 2018; Ibrahim et al., 2013; Kinman, 2001). Not being cited or recognized in ways deemed meaningful in the academy can have impacts on feelings of self-worth, self-confidence, job satisfaction, and heightened potential for burnout.

One of us has been employed as contingent and temporary faculty for the seven years following her PhD, and this experience has offered perspective on the lived realities of precariously employed academics. For instance, 25% of adjunct faculty in the US rely on public assistance and some make so little that they cannot afford housing and must live in their cars (Childress, 2019). These extreme and more mundane ways of living and working take their toll. Contingent faculty (a population that makes up 70% of US faculty positions [Curtis & Thornton, 2013]) suffer from particularly high rates of mental illness (Reevy & Deason, 2014). The contingent workforce, already deemed marginal demographically and professionally within the academy (even as they are increasingly the overwhelming majority), become further marginalized in citational structures. It is more difficult to maintain active research and publishing activities as a contingent faculty – both because of time constraints related to the need to take on higher teaching loads to make ends meet in poorly paid teaching positions with no benefits and because research funders tend to prioritize applicants from more prestigious and secure faculty positions (i.e. tenure-track positions or the equivalent). In contingent employment, and even in secure, tenure-track or equivalent positions, there is a dramatic inequity in who carries the burden of institutional service as uncompensated labor that takes valuable time away from research and publishing – Black, Indigenous and people of color (BIPOC) are disproportionately tasked with these forms of labor, including EDI efforts (Ahmed, 2012; Barcan, 2013; Gutiérrez y Muhs et al., 2012). In the politics of citation, this inequity plays out in citing or inviting to speak those scholars who, from places of institutional and socio-demographic privilege, have produced an abundance of scholarship, rather than those who may publish less as a result of institutional inequities.

### Citing others

We have been acutely aware that when we cite someone/something (including the citations in this paper!), we are perpetuating or (on rarer occasions) challenging social and knowledge structures and norms, and also indirectly affecting the career trajectories of others. At the same time, we have discovered that it is much easier to debate and theorize citational inequities, and advocate radical change, than it is to call out individual instances of problematic citation or to practice inclusive citation. Good intentions take one only so far, and intentions are not always straightforward. For instance, one of us, while trying to write on urban natures, quickly realized that those seen as ‘key’ scholars of urban nature are predominantly white and often male – and typically espousing certain onto-epistemological positions. These are the names that first come to one’s mind and that one hears and sees repeated again and again in peer review comments. These comments have included recommendations to engage with the work of Derrida and Esposito as necessary to substantiate arguments made about and from a location in the Global South, even while questioning the value of the analysis (because of the location) to urban natures in other parts of the world. In turn, this means that these gatekeeper names and associated onto-epistemologies must be included to pass peer review, regardless of their relevance or usefulness.

There are always exceptions, but they remain precisely that, and are often those who have somehow achieved a certain kind of ‘star’ status in their field. Or they are those who have made a name for themselves talking about issues relating to diversity in some form or the other. These exceptions quickly become the token names that must be cited to establish one’s inclusivity and avoid accusations of bias, and that thereby enable the sustenance of established norms and structures of knowledge. They become citationary alibis that ‘consolidate rather than challenge such forms of citationary asymmetry’ (Roy, 2020, p. 20). Furthermore, there is only limited space for such tokens, which engenders an unhealthy competition between those who could potentially be such tokens.

The sheer quantum of publishing makes it even more difficult for lesser-known scholars to gain visibility. We spend time trawling through databases and reference lists to find and read the work of lesser-known scholars in the hope of finding new inspiration and crafting more diverse bibliographies. But we do this with the nagging fear that we are spending much more time on our papers (and on our citations) than other more ‘productive’ colleagues would do, and that we may not be able to include most of these lesser-known names anyway because the word limit will be used up by gatekeeper names. Fatigue and a sense of futility start to override good intentions, as has happened even while putting this paper together.

How can we counter this fatigue and futility? How do we join the dots between discipline-level citational structures, wider debate on citational politics and everyday experiences and practices of scholarship? How do we tackle citational inequities within the imperfect academic spaces that we inhabit, instead of setting our sights on an imagined radically inclusive academy as the only goal? How can we craft, through our citation practices, more onto-epistemologically inclusive and diverse geographies?

As Mott and Cockayne (2017, p. 954) write, ‘[c]itation thought conscientiously can also be a feminist and anti-racist technology of resistance.’ In terms of practical steps, they suggest a close counting of citations prior to submission wherein the author learns about and notes who they have cited and what subject positions they reflect; a concerted effort made particularly by senior, established scholars to cite a wider range of scholarship and the work of more junior scholars; critical attention to citation practices by reviewers and editors during the peer review process; and encouragement and valuing of other forms of disseminating knowledge than the more formalized academic publication format.

### Enabling more diverse citation

In particular, citational practice can be transformed during both the peer review process and in formalized and informal education of graduate students. In our publishing experience, we have frequently encountered gatekeeping in terms of whose (and what) work we cite and engage. As authors, we have all had the experience of being instructed, by both reviewers and editors in the peer review process, to cite certain Big Names and Big Theories. It is not that we are unaware of who the Big Names or what the Big Theories are in our field; rather, we have often made explicit decisions *not* to cite them, either to make room for lesser-known scholars or simply because there are more relevant and/or more interesting works and approaches with which we wish to engage. The options, then, when confronted with demands to include these commonly cited ‘stars’ in the field are either to capitulate or to make the case to editors and reviewers that we do not need to cite these particular names and works again.

After a decade or more in our fields, we each feel somewhat more confident pushing back on reviewers and editors to explain why we may have chosen not to cite certain popular scholars and literatures. However, for those who may not feel as comfortable standing up for their decisions, we call on reviewers and editors to think about the powerful role that they hold in helping to encourage and shape a more diverse and inclusive scholarship – in not reflexively recommending/requiring the citation of specific ‘stars’ and specific works, and in encouraging authors to engage with other literatures when they might have prioritized the Big Names and Big Theories in the papers that they submit.

This same effort might be made more robustly in undergraduate, masters and doctoral education and advising; faculty might encourage students to explore a wider range of scholarship that routinely goes under-acknowledged, rather than requiring a persistent and disproportionate focus on the ‘stars’ in the field. This move could, in fact, require students more rigorously to develop and demonstrate their ability to conduct comprehensive literature reviews and better to understand the full breadth of their fields.

Another remedy might be simply to include a broader range of scholarship in our publishing practices by citing works that may be typically marginalized in the discipline. This is certainly a start, and, in the absence of a more comprehensive response, a good one. In the interim – between now and the successful overthrow of systems of epistemological oppression – more citation of those scholars as well as literatures that are under-cited or not cited at all as a result of their marginalization in academic scholarship is a necessary and powerful practice.

As Avril Maddrell (2012, p. 326) points out, however, ‘citation might be read as indicative of engagement, but as such that ‘engagement’ can be a very superficial one, one which acknowledges the existence of a body of work through name-checking, but which fails to attend to, disseminate, reinforce or critique the detail of that work.’ We should ask what *engagement* means in our writing and how it can reject the superficiality of the parenthetical citation. Engagement takes careful thinking across all stages of research and writing, beginning with what questions we ask and how we set out to answer them; it takes a willingness to be fundamentally changed and transformed by someone else’s work; and it takes *time.*

Each of us has had the experience of rushing to get an article written – we reflexively drop into the text those easy citations we have in our back pockets (e.g. *Timothy Pachirat writes on the exploitative nature of slaughterhouse labor. Cite him. Move on. Next sentence.*). These easily recalled citations are the ones that everyone cites (and they are easy to recall because everyone cites them). We are conscious of the time-consuming nature of trying to track down, read, and think with lesser-known scholarship given the pressures, quantum, and speed at which publishing is meant to occur in the neoliberal academy. Indigenous and feminist geographers have duly called for geographers to join the germinating interdisciplinary movement for ‘slow scholarship’ – an intentional slowing down to take ‘time to think, write, read, research, analyze, edit, and collaborate’ against the externally imposed pressures of ‘academic productivity’ measures (Mountz et al., 2015, p. 1237; also see Simpson, 2017). As Mountz et al. explain, ‘the ‘slow’ in slow scholarship is not just about time, but about structures of power and inequality’ (p.1238). For slow scholarship to take hold, it must be taken up as a restructuring of the academy writ large so that quality and variety is valued over the speed at which scholarship can be produced. Might the scholarship we generate as geographers be more thoughtful, more varied, and *just all around better* for having taken the time to read more widely, to think carefully about the practice of citation, and to allow what we have read to wash over us and change the way we think about and practice the work we are doing?

## Conclusion

In this paper, we examine how citation inequities operate within Anglophone animal geographies beyond axes of socio-demographic difference. Through a bibliometric analysis of animal geographies literature from 1998 to 2019, we highlight the citation politics of this subdiscipline-in-the-making. Our analysis is unavoidably partial; our method, using WoS data, excludes books and some articles and comes with limitations such as including self-citations – all of which inevitably shape what comes into view. Rather than regarding our results as a complete, final picture of citation in animal geographies, we see our them as a departure-point, an invitation for what we hope will be further examinations of citational politics in animal geographies and, ultimately, changed citational practices.

Our results, while preliminary and partial, do suggest a stark unevenness in Anglophone animal geographies’ citational practices, skewed towards UK-based institutions in particular (as opposed to other Anglophone countries dominating the top 25), as well as a privileging of certain themes and approaches. They suggest that institutional and locational networks, coupled with shared onto-epistemologies and ethical and political orientations, play a key role in shaping citation structures. These citational structures are reflective of the consolidation of specific types, and even pieces, of scholarship as the ‘state of knowledge’ in the subdiscipline, specifically work rooted in a small selection of Continental philosophers and relational approaches, and that refrains from explicitly anti-anthropocentric arguments, even while acknowledging the importance of Indigenous and non-mainstream perspectives.

This is not to say that other onto-epistemologies and political orientations are absent in animal geographies scholarship (for a recent review, see Narayanan, 2023). However, they remain in the margins of the field, with consequences for the overall contours of knowledge, as well as for individual academic trajectories. As we discussed in our exploration of everyday citational politics, citational structures can make it very challenging for individual scholars to engage in an academic practice that transgresses the limits established by ‘top’ scholars, ‘top’ works, and ‘important’ approaches. More importantly, the influence of citational trends and esteem has multispecies justice impacts by limiting the circulation and reach of scholarship that engages with the ethical and political conditions that (often adversely) affect nonhuman animals.

As animal geographies continues to grow and its scope expands, our hope is that so too will the space for a wider range of onto-epistemological approaches and ethical and political directions, especially those that explicitly tackle the uneven power relations and structural injustices that dominate the lives of animals in human society. Citation is just one path towards creating that space, but it is a concrete and near-everyday academic practice that subtly but influentially, through repetition, reproduces intellectual space. Engaging meaningfully in a more expansive citational practice can therefore powerfully reproduce that space otherwise.

## Note

1 The asterisk is a wildcard symbol that searches for all variations on a word (e.g. speciesis* searches for speciesism, speciesist).
